# A Leave of Absence Might Not Be a Bad Thing: Registered Practical Nurses Working in Home Care During the COVID-19 Pandemic

**DOI:** 10.1177/10848223241232408

**Published:** 2024-03-16

**Authors:** Denise M. Connelly, Nicole A. Guitar, Anna Garnett, Tracy Smith-Carrier, Kristin Prentice, Jen Calver, Emily King, Sandra McKay, Diana Pearson, Samir Sinha, Nancy Snobelen

**Affiliations:** 1 Western University, London, ON, Canada; 2 Royal Roads University, Victoria, BC, Canada; 3 Ontario Tech, Oshawa, ON, Canada; 4 VHA Home Health Care, Toronto, ON, Canada; 5 Lambton College, Sarnia, ON, Canada; 6 University of Toronto, Toronto, ON, Canada; 7 Registered Practical Nurses Association of Ontario, Chatham-Kent, ON, Canada

**Keywords:** survey, nurses, home care services, resilience, emotional intelligence, COVID-19

## Abstract

To describe the resilience and emotional intelligence of Registered Practical Nurses working in Home and Community Care during the COVID-19 pandemic. Specifically, to determine if there was a relationship between resilience and emotional intelligence based on whether a nurse: (1) left the sector, (2) considered leaving, or (3) took a leave of absence during the pandemic. An online cross-sectional survey was used to capture respondents’ demographic information and scores on the Connor–Davidson Resilience Scale, Resilience at Work Scale^®^, and Wong and Law Emotional Intelligence Scale. Registered Practical Nurses working, or who had worked, in Home and Community Care January 2020 to September 2022 were eligible to participate. The Checklist for Reporting Results of Internet E-Surveys was used. The survey was available June to September 2022 and advertised by the Registered Practical Nurses Association of Ontario to approximately 2105 members. Descriptive statistics and independent samples *t*-tests were used to analyze results at a level of *P* < .05 was used for all analyses. A total of 672 respondents participated (completion rate = 92.8%). There were no differences on resilience or emotional intelligence scores based on whether a nurse left, or considered leaving, the Home and Community Care sector during the pandemic. However, nurses who took a leave of absence scored significantly higher on resilience and emotional intelligence measures when compared to those who did not. Results suggest that a leave of absence for these nurses during the pandemic may have been a supportive coping strategy.

## Introduction

Home and Community Care (HCC) nurses have faced a multitude of challenges in recent years; difficulties that were compounded during the Coronavirus disease 2019 (COVID-19) pandemic. HCC services provide care to people in their home, at school or in the community.^1^ The global demand for community-based care has been intensifying over time, increasing pressure on this already strained workforce.^2^ Ongoing changes in the structure and function in HCC services can create fluctuations in the nursing labor force.^2^ Nursing shortages, which pre-date the pandemic, are occurring across all health care sectors due to excessive workloads and job demands that lead nurses to feel disrespected, frustrated, overwhelmed, and burnt out.^3,4^ In addition, our global aging population is associated with an increase in aging-related health conditions and comorbidities that may necessitate nursing care.^5-7^

HCC nurses are leaving the workforce in large numbers since the pandemic, weakening a sector that has been chronically understaffed, and placing immense pressure on the nurses who remain to manage growing caseloads with insufficient support.^8^ Throughout the pandemic, nurses in all sectors faced the risk of contracting COVID-19, often without sufficient access to Personal Protective Equipment (PPE), or adequate training,^9^ Although infection rates in HCC were lower than in institutional settings,^10^ this was a source of considerable concern for HCC nurses who continued to enter client homes and in some cases risk exposure during transit.^9^ Nurses also face numerous stressors, conflicts, and ethical dilemmas that can intensify moral distress and require immense emotional intelligence to navigate.^11-13^ Resilience has been suggested to be helpful in enabling nurses to continue working through a range of challenging conditions but to date we have limited knowledge of any relationship(s) between resilience and emotional intelligence based on whether a nurse: (1) left the sector, (2) considered leaving, or (3) took a leave of absence during the pandemic.

### Case Example: Registered Practical Nurses (RPNs) in Ontario

RPNs are one of 2 categories of the regulated nursing profession in Ontario who are members of the College of Nurses of Ontario (CNO) and legislated under the *Nursing Act (1991)* and *Regulated Health Professions Act (1991)*.^14^ Of the approximately 60,000 RPNs working in Ontario, over 13,000 are currently employed in community health settings, including 3529 in HCC.^14^ Some nurse titles internationally that have a similar scope to Ontario RPNs include Licensed Practical Nurses (LPNs)^15-17^ in other Canadian provinces, Enrolled Nurses (ENs)^18,19^ in Australia and New Zealand, and Assistant Practitioners, Associate Degree and Associate Nurses (AP, ADN, AN)^20,21^ in the United Kingdom.

### Theoretical Frameworks

Resilience and emotional intelligence have been individually linked with nurse retention.^22,23^ Rushton et al^24^ found that resilience can act as a protective factor against stress, burn out, and emotional exhaustion for nurses. Emotional Intelligence (EI) has also been shown in the literature to promote mental health in nurses and help them better cope with stress.^25^

### Emotional Intelligence

EI includes “a set of interrelated skills featuring the ability to perceive accurately, appraise, and express emotion. . . and the ability to regulate emotions to promote emotional and intellectual growth.”^27(p10)^ EI allows nurses to form and sustain positive relationships “in emotionally charged environments where emotion is central to the fabric of health care delivery.”^(p1625)^ The decisions that nurses must continuously make in their clinical practice, bound by codes of practice and their professional ethics, occur in environments characterized by chaos and change. Internationally, EI has been shown to have a significant positive relationship with resilience for nurses^28,29^ and nursing students.^30^

### Resilience

Resilience is a critical factor in enabling nurses to continue to practice under the challenging conditions that frequently characterize their work, particularly in underserviced sectors.^31^ While *personal resilience* captures the processes by which people “bounce back” from adversity or misfortune using biological and psychological strengths that enable them to cope with threats and challenges as they arise, *professional resilience* (i.e., resilience at work) refers to the ability of individuals to thrive in the context of challenging environments.^32^ Nurses demonstrating resilience at work experience protective effects and are better able to cope with stressors and engage resources that optimize their well-being.^44^

### Aim

The aim of the study was to describe the resilience and EI of Registered Practical Nurses working in HCC during the COVID-19 pandemic and to determine if there was a relationship between resilience and EI based on whether a nurse: (1) left the sector, (2) considered leaving, or (3) took a leave of absence during the pandemic.

## Method

### Ethics

Ethical approval for the study was obtained from the XX, Institutional Review Board (#XX) in XX.

### Survey Study Design and Development

Qualtrics XM (Provo, UT) software was used as the platform for an open online survey. The survey could be accessed and completed using a computer or smartphone and was accessible in English only between June 10th to September 7th, 2022. Responses were securely stored on a firewall protected computer. The survey contained a total of 12 pages and the number of responses required by respondents per page ranged from 1 to 46 when each item on a Likert-scale is counted as a unique response. A total of 33 questions were presented to respondents (some of which contained Likert scales with up to 11 items each). Respondents were given the option to navigate backward in the survey, to skip questions, not provide a response to a question, and to pause and return to the survey later. Adaptive questioning was used only for questions that necessitated a response based on a previous one. There was no time cut-off for the completion of the survey.

An incentive was provided to participants such that if they completed the survey, they could choose to be entered into a random draw to receive one of 500 $25 gift cards for a recognized grocery chain. Electronic gift cards were sent to respondents via email. Bot-detection software on Qualtrics was used to detect suspected bots and to remove potentially fraudulent responses. The study team reserved the right to not send gift cards to email addresses that were suspected to be fraudulent.

Survey items included multiple choice, Likert scales, and Yes/No questions. The cross-sectional survey collected descriptive information about nursing tenure, employment status, satisfaction with their employment during COVID-19, whether they took a leave of absence or left the HCC sector during the pandemic (and if so, their reasons for leaving), their COVID-19 infection history, perceptions of their supervisor(s)’ and employers’ communication during the pandemic, their physical and mental health, and demographic characteristics including age, gender, marital status, ethnicity, citizenship, and income. In addition, respondents were asked to complete 3 assessment measures: the Connor–Davidson Resilience Scale (CD-RISC-10),^33^ the Resilience at Work Scale^®^ (R@W),^34^ and the Wong and Law Emotional Intelligence Scale (WLEIS^35^; see Table 1 for descriptions of the included assessment measures).

**Table 1.** Included Outcome/Assessment Measures.

Assessment measure	Description
Connor–Davidson Resilience Scale (CD-RISC-10)	The CD-RISC-10 is a unidimensional scale that allows for an efficient measurement of resilience. It has good internal consistency (Cronbach’s alpha = 0.85) and construct validity. The items in the CD-RISC-10 reflect the ability to tolerate experiences such as change, personal problems, illness, pressure, failure, and painful feelings (Campbell-Sills & Stein, 2007). The scale measures six components of resilience including the ability to: (1) adapt to change, (2) deal with what comes along, (3) cope with stress, (4) stay focused and think clearly, (5) not get discouraged in the face of failure, and (6) handle unpleasant feelings such as anger, pain, and/or sadness (Petzel, 2021).^36^ The CD-RISC-10 has been found to show the best combination of reliability, validity, and practicality for measuring resilience when compared to other longer versions of Connor & Davidson’s resilience scales (e.g., CD-RISC-25; Kuiper et al 2021).^37^ The CD-RISC-10 contains 10 items that are rated on a 4-point Likert scale ranging from “not true at all” (0) to ‘true nearly all the time’ (4). Total CD-RISC-10 scores range from 0 to 40, with higher scores indicating greater personal resilience (Campbell-Sills & Stein, 2007). No cut-off exists to indicate the presence of resilience based on the CD-RISC-10; however, data from 764 participants provides quartile ranges (scores of 0-29 = first, 30-32 = second, 33-36 = third, and 37-40 = fourth; Campbell-Sills & Stein, 2007; Davidson, 2018). Permission to use the CD-RISC-10 was obtained by a study team member via email from Johnathan Davidson on June 9, 2022.
Resilience at Work Scale® (R@W)	The R@W scale was used to measure respondents’ personal resilience, in the context of their workplace specifically. The R@W scale is a reliable 20-item tool that measures seven domains of resilience in the context of work through seven subscales (i.e., Living Authentically, Finding Your Calling, Maintaining Perspective, Managing Stress, Interacting Cooperatively, Staying Healthy, and Building Networks; McEwen, 2019; Winwood et al, 2013). Each item on the scale is rated on a seven-point Likert scale ranging from “strongly disagree” (0) to “strongly agree” (6) with two items reverse-scored. Higher total and subscale scores are indicative of higher resilience (possible scores range from 0-120; Winwood et al, 2013).
The Wong and Law Emotional Intelligence Scale (WLEIS)	The WLEIS provides a short measure of EI (i.e., one’s self-reported capacity to examine and control emotions) that is suitable for research in the workplace (Wong & Law, 2002). The internal consistency of the WLEIS is high (i.e., Cronbach’s alpha ranges from 0.83-0.90; Wong & Law, 2002). The WLEIS also has acceptable reliability, convergent and discriminant validity in healthcare professionals specifically (Shah, 2022). The scale contains 16 items that are scored on a 7-point Likert-scale ranging from “strongly disagree” (1) to “strongly agree” (7). The total score can therefore range from 7 to 112. Lower scores indicate lower EI, while higher scores indicate higher EI.

### Sample & Recruitment

Only RPNs who worked in HCC in Ontario January 2020 to September 2022 during the COVID-19 pandemic were eligible to participate in the study, regardless of their current employment.

Respondents were recruited through their professional association, the Registered Practical Nurses Association of Ontario (WeRPN). WeRPN sent, over a 3-month period, a series of email invitations that included the online survey link to approximately 2105 potential respondents currently documented as working in HCC in Ontario. Postings for the online survey were also advertised through WeRPN’s newsletter and their social media channels (e.g., Facebook, Instagram). A reminder email from WeRPN was sent 2 weeks after the initial invitation to encourage participation, as recommended by Sammut et al.^38^ No direct contact was made with potential respondents and survey responses were anonymous. The collection of additional system data (e.g., respondents’ IP addresses) was disabled using Qualtrics software, which uses encryption technology and restricted access authorizations to protect all data collected. No other log file analyses were used. The use of non-probabilistic sampling, due to the physical and fiscal constraints of obtaining province-wide access to individual contact information, prevented the calculation of a participation or view rate (i.e., we are unable to determine how many eligible people were exposed to our invitation to participate).^39,40^ Informed consent to participate was obtained on the landing page of the online survey.

### Data Management and Statistical Analyses

Survey data were exported from Qualtrics and organized within Excel software. Data analyses were completed using SPSS Version 29 (IBM). It was determined a priori that only questionnaires that were≥ 90% complete would be analyzed. Descriptive statistics were run, and any missing data from responses that were between 90% and 100% complete was excluded in the descriptive statistic calculations. Independent samples *t*-tests were used to determine if there were significant differences between scores on CD-RISC-10, R@W or WLEIS for respondents who (1) left HCC during the pandemic, (2) reported they considered leaving HCC during the pandemic, and (3) took a leave of absence from HCC during the pandemic, when compared to those who did not. Lastly, independent samples *t*-tests were inspected to examine if there was a significant difference between self-reported physical and mental health prior to and during the COVID-19 pandemic. For all statistical tests, alpha was set to <.05.

### Research Reporting Checklist

The Checklist for Reporting Results of Internet E-Surveys (CHERRIES) was used (see Appendix A).^41^

## Results

A total of 768 RPNs consented to participate in the survey; however, 10 respondents did not describe themselves as an RPN working in HCC, and another 10 reported that they did not work during the COVID-19 pandemic and were therefore excluded from the analysis. Lastly, 24 responses were removed either manually or by Qualtrics bot detection software as suspected fraudulent responses, (i.e., those that used an email address identical to one used by another respondent, but with a different number at the end). Of the remaining 724 responses, 52 surveys were <90% complete and were therefore excluded. Accordingly, the total number of eligible respondents who completed the survey was n = 672 (completion rate of survey = 92.8%; see Table 2 for a description of participants’ ages and experience in HCC and Table 3 for further descriptive characteristics). The mean and median time for completion of the survey was 15.44 minutes (*SD* = 40.6 minutes) and 7.34 minutes, respectively.

**Table 2.** Description of RPNs Age and Experience in HCC.

	N	Mean	SD	Min-Max
Age (years)	669	38.02	8.05	23.00-64.00
Years registered as a nurse	672	13.27	5.86	3.00-40.00
Years worked in HCC	672	6.83	4.58	1.00-31.00
Hours worked per week in HCC	672	27.20	12.68	2.00-75.00

**Table 3.** Description of RPN Respondents.

Demographic characteristic	N	Percentage of respondents
Marital status		
°Married/common-law	510	75.9%
°Separated/divorced	81	12.1%
°Single	64	9.5%
°Widowed	17	2.5%
Gender		
°Woman	334	49.7%
°Man	329	49.0%
°Additional identities	9	1.2%
Status in Canada		
°Canadian citizen	308	45.8%
°Permanent resident	296	44.0%
°Temporary resident	66	9.8%
°Other	2	0.3%
Ethnicity		
°White/Caucasian	397	59.1%
°Black	92	13.7%
°South Asian	77	11.5%
°Latin American	47	7.0%
°Southeast Asian	23	3.4%
°West Asian	14	2.1%
°Indigenous: First Nations	5	0.7%
°Chinese	5	0.7%
°Korean	5	0.7%
°Filipino	4	0.6%
°Other	1	0.1%
°Japanese	1	0.1%
Employment status		
°Full-time RPN	370	55.1%
°Part-time (1 or more positions)	211	31.4%
°Both of the above	91	13.5%
LHIN		
°Central West	78	11.6%
°Toronto Central	75	11.2%
°Waterloo Wellington	65	9.7%
°Southwest	65	9.7%
°Mississauga Halton	63	9.4%
°Hamilton Niagara Haldimand Brant	59	8.8%
°Central	44	6.5%
°Erie St. Clair	44	6.5%
°Central East	38	5.7%
°North Simcoe Muskoka	36	5.4%
°Southeast	35	5.2%
°Champlain	34	5.1%
°Northeast	26	3.9%
°Northwest	10	1.5%
Employment in another healthcare sector in addition to HCC		
°Yes	494	73.5%
°No	178	26.5%
Considered leaving HCC		
°No	492	73.2
°Yes	180	26.8
Left HCC		
°Yes	112	16.7
°No	43	6.4
°Temporarily	25	3.7
Income change during COVID-19		
°Increased	294	43.8
°Stayed the same	195	27.2
°Decreased	183	29.0
Received income supplements		
°Yes	441	65.6
°No	231	34.4
Took a leave of absence during COVID		
°No	385	57.3%
°Yes	287	42.7%
Top reason reported for leave of absence		
°Family-related responsibilities	74	11.0%
°Employment Policy	53	7.9%
°Personal health-related reasons	113	16.8%
°Fear of COVID-19	27	4.0%
RPN report of whether they ever tested positive for COVID-19		
°Yes	284	42.3%
°No	388	57.7%

*Note.* Gender “other” includes: Non-binary (n = 1), genderqueer (n = 4), third gender (n = 1), 2-spirit (n = 1) and prefer not to say (n = 2).

**Figure 1.**
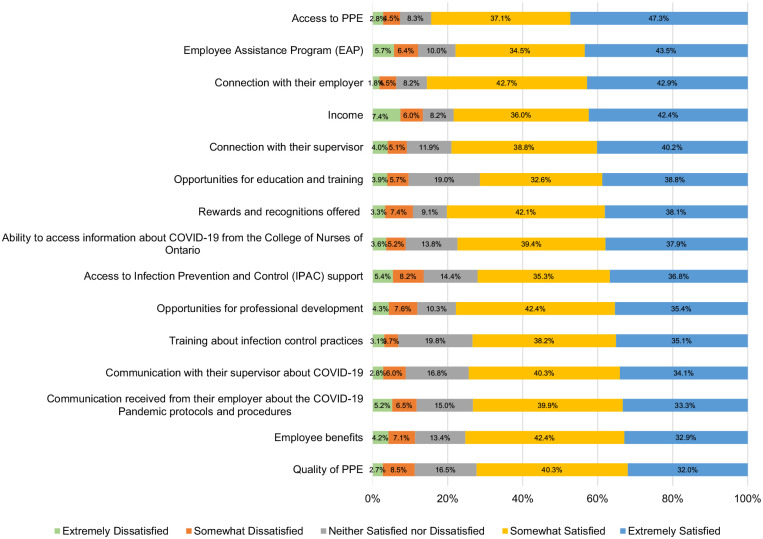
Percentage of RPNs’ satisfaction with various HCC workplace factors during COVID-19 (n = 672).

**Figure 2.**
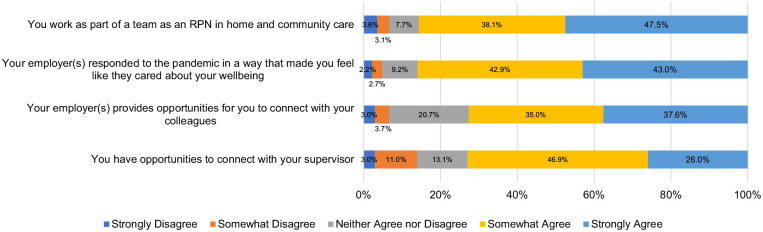
Percentage of agreeance about working in HCC during COVID-19 (n = 672).

**Figure 3.**
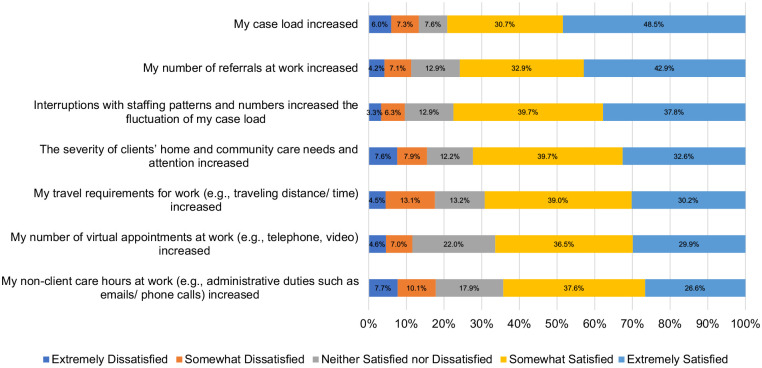
Percentage of agreeance with statements about HCC work changes during COVID-19 ( n = 672).

**Figure 4.**
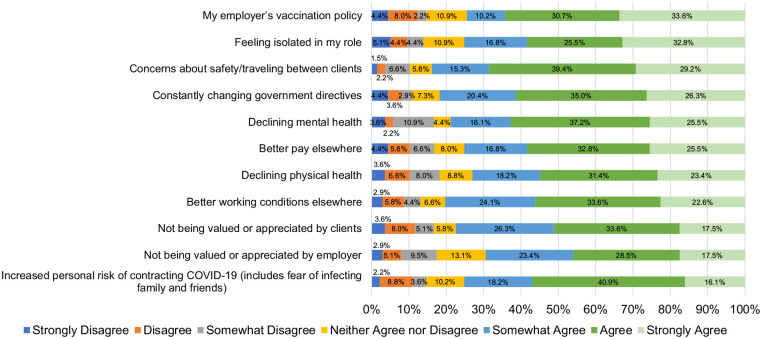
Reasons RPN’s left the HCC sector during COVID-19 (n = 137).

Group mean score for respondents was 28.8/40 on the CD-RISC-10 (SD = 5.52, min, max = 9, 40), indicating that their resilience was low (i.e., in the first quartile of the average/nominal score distribution).^42,43^ Scores on the R@W scale are presented both as Likert-scale means and as standardized R@W scores, as indicated in the Resilience at Work^®^ Manual (see Table 4).^44^ On the R@W scale, responses were average (i.e., 71%) when compared to standardized scores in the McEwen Resilience at Work^®^ Manual. Table 5 shows scores on the WLEIS. RPNs’ reported satisfaction with a range of HCC workplace factors is available on Figure 1. Respondents’ agreement with various statements about working in HCC during COVID-19 is available on Figure 2. Further, RPNs’ reported agreeance with statements about changes to their work in HCC during COVID-19 is available on Figure 3. For those RPNs who reported that they had left the HCC sector during COVID-19 (n = 137), the reasons for leaving are reported on Figure 4. The top reasons RPNs reported for taking a leave of absence in the present study in descending order were: “their employers vaccination policy,” “feeling isolated in their role” and “concerns about their safety.”

**Table 4.** Group Data for RPN Scores on the R@W Scale.

	N	Likert-scale mean (/6)	Likert-scale SD	Standardized mean (%)	SD (%)	Min-Max (%)
R@W total	672			71.75	11.32	22.50-90.83
R@W subscales						
Building networks	672	4.69	1.10	78.10	18.38	0.00-100.00
Staying healthy	672	4.58	1.14	76.36	19.02	0.00-100.00
Living authentically	672	4.56	1.00	76.03	16.62	11.11-100.00
Interacting cooperatively	672	4.54	1.10	75.63	18.39	0.00-100.00
Managing stress	672	4.47	1.01	74.46	16.80	12.50-100.00
Finding your calling	672	4.45	0.97	74.11	16.12	8.33-100.00
Maintaining perspective	672	3.05	1.23	50.84	20.56	5.56-100.00

*Note.* R@W: Resilience at Work Scale (Winwood, Colon & McEwen, 2013); Standardized scores are Likert-scale scores converted according to the Resilience at Work (0 = strongly disagree, 6 = strongly agree) Research Manual (McEwen, 2019); Scores of 0 in the Min-Max column indicate Likert scores of 0 (strongly disagree) converted to percentages (i.e., at least one respondent indicated they strongly disagreed to items in that subscale). Nurses were most able to develop their capacity to Build Networks (i.e., develop and maintain workplace and personal support networks), followed by Staying Healthy (i.e., maintaining energy through a good level of physical fitness and a healthy diet), Living Authentically (i.e., maintain personal values, use personal strengths, and have good emotional awareness and regulation at work), Interacting Cooperatively (i.e., seeking feedback, advice and support, and providing support to others), Managing Stress (i.e., maintain work life balance, engage in relaxation, and use work and life routines that help manage everyday stressors), Find their Calling (i.e., seeking work that has a purpose, gives a sense of belonging and fits well with one’s core beliefs), and finally, Maintain Perspective (i.e., manage negativity, reframe difficulties and setbacks, and focus on solutions at work).

**Table 5.** Group Data for RPN Scores on the WLEIS.

	N	Likert-scale mean (/7)	Likert-scale SD	Min-Max (%)
WLEIS total intelligence	671	5.59	0.98	1.75-7.00
Others-emotion appraisal	672	5.67	0.92	1.75-7.00
Use of emotion	671	5.59	0.98	1.75-7.00
Self-emotions appraisal	672	5.58	0.95	1.50-7.00
Regulation of emotions	672	5.45	0.99	1.75-7.00

*Note.* WLEIS: Wong and Law Emotional Intelligence Scale (Law, Wong & Song, 2004; 1 = strongly disagree, 7 = strongly agree).

Independent-samples *t*-tests were run to determine if there were differences on CD-RISC-10, R@W, and/or WLEIS scores based on whether an RPN reported that they (1) had left the HCC sector during the pandemic; (2) had considered leaving the HCC sector during the pandemic, or (3) took a leave of absence from the HCC sector during the pandemic (Table 3). There were no significant differences between scores on any of the 3 scales based on whether nurses had (1) left the HCC sector during the pandemic or (2) considered leaving the HCC sector during the pandemic.

When stratifying the sample according to whether the participant took a leave of absence or not from the HCC sector during the pandemic, significant differences in scores were found on each of the CD-RISC-10, R@W and WLEIS. There were no outliers in the data, as assessed by inspection of a boxplot. Scores for the CD-RISC-10, R@W and WLEIS were normally distributed, as assessed by Shapiro-Wilk’s test (*p* > .05), but the assumption of homogeneity of variances was violated, as assessed by Levene’s test for equality of variances (*p* < .001 for CD-RISC-10, R@W and WLEIS). Scores were higher on CD-RISC-10, R@W, and WLEIS for those who had taken a leave of absence (*M* = 29.96, *SD* = 4.65, *M* = 73.65, *SD* = 10.07, and *M* = 5.79, *SD* = 0.72, respectively) as compared to those who had not (*M* = 28.05, *SD* = 5.95, *M* = 70.33, *SD* = 11.98, and *M* = 5.40, *SD* = 0.81, respectively) with a statistically significant difference on each of the CD-RISC-10, *M* = 1.90, 95% CI [1.10, 2.70], *t*(668.41) =4.65, *p* < .001, R@W scale *M* = 3.32, 95% CI [1.65, 4.99], *t*(660.38) = 3.91, *p* < .001, and WLEIS *M* = 0.39, 95% CI [0.27, 0.50], *t*(647.73) = 6.54, *p* < .001.

Additional paired-samples *t-*tests were used to determine whether there were statistically significant differences between self-reported mental and physical health prior to and during COVID-19. Two outliers were detected that were more than 1.5 box-lengths from the edge of the box on a boxplot. Inspection of their values did not reveal them to be extreme and they were kept in the analysis. Mean mental health was higher prior to COVID-19 (*M* = 2.14, *SD* = 1.05) when compared to during COVID-19 (*M* = 2.10, *SD* = 1.04), with a non-statistically significant difference of −0.03, 95% CI, [−0.12, −0.05], *t*(670) = −0.78, *p* = .218, *d* = 1.13. Physical health was also higher prior to the COVID-19 pandemic (*M* = 2.08, *SD* = 1.00) as compared to during the pandemic (*M* = 2.10, *SD* = 1.04), with a statistically significant difference of −0.13 95% CI [−0.21, −0.05], *t*(671) = −3.24, *p* < .001, *d* = 1.04.

## Discussion

In the present study, an open cross-sectional online survey was used to describe the resilience of RPNs (n = 672) working in HCC during the COVID-19 pandemic. The CD-RISC-10, R@W and WLEIS scales were included in the survey. RPNs scored as having low resilience on the CD-RISC-10 which may suggest problems with respondents’ ability to tolerate experiences such as change, personal problems, illness, pressure, failure, and painful feelings.^45,46^ On the R@W scale, scores were average with RPNs being most able to develop their capacity to Build Networks (i.e., develop and maintain workplace and personal support networks) and least able to Maintain Perspective (i.e., manage negativity, reframe difficulties and setbacks, and focus on solutions at work) as measured by the R@W subscales. There were no differences on CD-RISC-10, R@W, and/or WLEIS scores based on (1) whether an RPN reported they had left or remained within the HCC sector during the pandemic, or (2) whether an RPN reported they had considered leaving the HCC sector during the pandemic. However, those RPNs who reported that they took a leave of absence from the HCC sector during the pandemic scored significantly higher on all 3 assessment measures when compared to those who did not, suggesting that RPNs who had taken a leave had higher resilience and EI.

These findings align with previous literature suggesting that breaks at work are beneficial to nurses.^23-26^ The results of the current study; however, are the first to highlight the significant differences in both resilience and EI between nurses who did, and who did not, take a leave of absence during a prolonged healthcare crisis (i.e., COVID-19). In addition, previous research suggests that EI buffers the effects of negative emotions on job burnout in nurses,^47^ indicating that EI training could be implemented to help prevent the adverse effect of negative emotions at work on job burnout. As previously stated, higher scores on measures of resilience and EI have both been individually linked with increased nurse retention.^22,23^ Further, resilience acts as a protective factor against stress, burn out, and emotional exhaustion for nurses,^24^ and EI promotes mental health in nurses and help them better cope with stress.^25^ Therefore, the findings of this study suggest that taking a leave of absence may be beneficial for retaining nurses and promoting good coping mechanisms during a health crisis. Future research should endeavor to explore whether this applies to different members of interprofessional healthcare teams.

Like other healthcare sectors, results of the present study also suggest that nurses were feeling isolated in their roles in HCC during COVID-19. Similarly, in institutional long-term care, loneliness and isolation during the pandemic are well documented amongst nurses.^48^ Previous research shows that the duration of mask wearing is correlated with a nurse’s stress level, such that the longer one wears a mask, the higher their self-reported stress.^49^ This suggests that a higher frequency of breaks may mitigate this effect.^49^ In addition, use of PPE ≥4 hours at a time is associated with several physical problems for nurses including skin irritation, dry mouth, sweating, and headaches.^50^ These results indicate that pandemic preparedness including quality PPE and adequate staffing to ensure breaks during shifts can be used is a priority during a healthcare crisis.

### Limitations

While the results indicate that nurses who took a leave of absence from HCC during the COVID-19 pandemic had higher resilience and EI, these findings may not be generalizable to other contexts, countries, and other types of nurses. Some international nurse titles that have similar nursing practice to the RPN in Ontario include Licensed Practical Nurses, Enrolled Nurses, Assistant Practitioners, Associate Degree, and Associate Nurses. These titles may not necessitate the same level of nursing education and registration requirements as those specified for Ontario’s RPNs, and may therefore not reflect the autonomous responsibilities as RPNs. Further replication of these results internationally is required to be able to make this generalization about nurses.

## Conclusion

An open cross-sectional survey was used to describe the resilience and EI of RPNs working in HCC during COVID-19. RPNs who reported taking a temporary leave of absence during the pandemic were found to have significantly higher resilience and EI when compared to nurses who did not take a leave of absence. Consideration of the various reasons that underpin taking a leave of absence provide opportunities for organizations how to best support RPNs with the right supports for their healthy engagement and participation. Further, results suggest that respecting that, for some RPNs, time away from work may be a healthy part of their personal coping strategies.

## Supplemental Material

sj-docx-1-hhc-10.1177_10848223241232408 – Supplemental material for A Leave of Absence Might Not Be a Bad Thing: Registered Practical Nurses Working in Home Care During the COVID-19 Pandemic Supplemental material, sj-docx-1-hhc-10.1177_10848223241232408 for A Leave of Absence Might Not Be a Bad Thing: Registered Practical Nurses Working in Home Care During the COVID-19 Pandemic by Denise M. Connelly, Nicole A. Guitar, Anna Garnett, Tracy Smith-Carrier, Kristin Prentice, Jen Calver, Emily King, Sandra McKay, Diana Pearson, Samir Sinha and Nancy Snobelen in Home Health Care Management & Practice
